# Variations in Oral Microbiota Composition Are Associated With a Risk of Throat Cancer

**DOI:** 10.3389/fcimb.2019.00205

**Published:** 2019-07-03

**Authors:** Lili Wang, Gaofei Yin, Ying Guo, Yaqi Zhao, Meng Zhao, Yunyun Lai, Pengcheng Sui, Taiping Shi, Wei Guo, Zhigang Huang

**Affiliations:** ^1^Beijing Cheer Land Biotechnology Co., Ltd., CL Investment Group, Beijing, China; ^2^Department of Otolaryngology Head and Neck Surgery, Beijing Tongren Hospital, Capital Medical University, Beijing, China

**Keywords:** throat cancer, early diagnosis, oral health, microbiome, 16*S* rRNA gene, next-generation sequencing

## Abstract

In this study, a next-generation sequencing strategy on 16*S* ribosomal RNA (16*S* rRNA) gene was employed to analyze 70 oral samples from 32 patients with throat cancer, nine patients with vocal cord polyp, and 29 healthy individuals (normal controls). Using this strategy, we demonstrated, for the first time, that the salivary microbiota of cancer patients were significantly different from those of patients with a polyp and healthy individuals. We observed that the beta diversity of the cancer group was divergent from both the normal and polyp groups, while alpha-diversity indices such as the Chao1 estimator (*P* = 8.1e-05), Simpson (*P* = 0.0045), and Shannon (*P* = 0.0071) were significantly reduced in cancer patients compared with patients containing a polyp and normal healthy individuals. Linear discriminant analysis (LDA) and Kruskal–Wallis test analyses and real-time quantitative polymerase chain reaction (qPCR) verification test revealed that the genera *Aggregatibacter, Pseudomonas, Bacteroides*, and *Ruminiclostridium* were significantly enriched in the throat cancer group compared with the vocal cord polyp and normal control groups (score value >2). Finally, diagnostic models based on putatively important constituent bacteria were constructed with 87.5% accuracy [area under the curve (AUC) = 0.875, 95% confidence interval (CI): 0.695–1]. In summary, in this study we characterized, for the first time, the oral microbiota of throat cancer patients without smoking history. We speculate that these results will help in the pathogenic mechanism and early diagnosis of throat cancer.

## Introduction

Throat cancer is a type of head and neck carcinoma. This cancer type can be subcategorized into cancer of the oropharynx, hypopharynx, nasopharynx, and larynx depending on where the cancer initially occurred (Edwards and Mendes, 2011). Hypopharyngeal cancer has the highest mortality rate among all the head and neck carcinoma types. The latter cancer has one of the worst prognoses of any malignant head and neck cancer, and the 5-year survival rate is ~15–65% (Edwards and Mendes, 2011; Krstevska et al., 2012; Garden et al., 2016). The 5-year survival rate for early-stage (T1–T2) patients is ~60%, while an overall survival rate of only 17–32% is observed for late-stage patients (Zeng et al., 2010). Furthermore, hypopharyngeal cancer has the highest rate of metastases, the earliest incidence of nodal metastases, and the highest rate of distant metastases (Elias et al., 1995; Johansen et al., 2000). Unfortunately, throat cancers, especially hypopharyngeal cancer, are difficult to detect at the early stages, and by the time the disease is diagnosed, it is usually at an advanced stage (Elias et al., 1995; Johansen et al., 2000; Garden, 2001). Thus, research into the mechanisms that underpin throat cancer and strategies that facilitate the detection of specific biomarkers for early prevention and diagnosis is urgent.

Use of tobacco and (or) alcohol are the highest risk factors for throat cancer, with ~85% of patients having a history of tobacco usage (Anantharaman et al., 2011). Patients with laryngeal cancer and a history of tobacco usage have a 20% higher mortality incidence than laryngeal cancer patients with no history of smoking (Maier et al., 1992). Additionally, infection with human papilloma virus (HPV) is another factor that contributes to throat carcinogenesis (Fakhry et al., 2006). However, the association between HPV and throat cancer has been less well-studied (Mineta et al., 1998; Joo et al., 2014).

The relationship between microbiota composition and cancer occurrence has been extensively studied in recent years. Studies have revealed that oral, intestinal, gastric, and throat mucosal layers are colonized by human commensal bacterial populations, which play important roles in human health (Shin et al., 2017; Dejea et al., 2018; Gao L. et al., 2018). Variations in microbiota diversity and abundance have been shown to be associated with cancer (Tözün and Vardareli, 2016; Yang et al., 2018). *Helicobacter pylori* infection is an important pathogenic risk factor contributing to the development of gastric cancer (Correa et al., 1975; Lee et al., 2010; Maldonado-Contreras et al., 2011). *Helicobacter pylori* infection can lead to gastric dysbiosis, which induces cancer development (Bruno et al., 2018). In addition, changes in the gut microbiota are also associated with the development of colorectal carcinogenesis (Nakatsu et al., 2015). The relative pathogenic microbiome has been identified and validated (Amitay et al., 2017; Wong et al., 2017; Chung et al., 2018), and these microbial biomarkers can be used as an aid for early diagnosis of colorectal cancer. Interestingly, the oral microbiota may be diagnostic indicators of oral cancer (Mager et al., 2005). The saliva bacteria *Porphyromonas gingivalis, Fusobacterium nucleatum*, and *Prevotella intermedia* have been reported to significantly associate with oral squamous cell carcinoma (Mager et al., 2005; Gholizadeh et al., 2016). Besides, Gong et al. (2017) observed that an imbalance in the throat microbiota profile can increase the risk of laryngeal cancer (Gong et al., 2017). Hayes et al. (2018) demonstrated that the relative abundance of oral bacteria including *Corynebacterium* and *Kingella* was negatively associated with the occurrence of laryngeal cancer (Hayes et al., 2018). Although little has been studied, previous studies indicated that the oral bacterium may play an essential role in throat carcinogenesis. Further studies need to be done to confirm the relationship between the human microbiome and throat cancer.

In this study, we investigated the link between the oral microbiome and throat cancer while also attempting to identify bacterial taxa that might represent risk factors in relation to throat carcinoma. We hope that these results provide a platform to further investigate the pathogenic mechanism of oral bacteria for throat cancer.

## Patients and Methods

### Patients

The study was approved by ethics committee of Beijing Tongren Hospital, Capital Medical University (approval number TRECKY2018-007). All participants upon recruitment signed informed consent. A total of 70 subjects were enrolled from April 17, 2017, to January 12, 2018, including 32 throat cancer patients, nine vocal cord polyp patients, and 29 healthy controls (sample information is in Supplementary Table 1). The individuals in the throat cancer and vocal cord polyp groups were between 45 and 71 years of age; the control group was assembled with individuals of similar age. The majority of the cases were male due to disease morbidity (Gale et al., 2010). All recruited participants had no history of tobacco or alcohol use. Subjects who used antibiotics in the preceding 6 months were excluded from the study.

Saliva specimens were collected from subjects in Beijing Tongren Hospital by using the Oragene DNA Sample Collection Kit (OG-500), which permits saliva specimens to be preserved for years at room temperature without DNA degradation. A volume of 2 ml of saliva was collected from each subject and mixed well with the Oragene solution. All samples were stored at −80°C for further analysis.

### DNA Extraction and Sequencing

Oral bacterial genomic DNA was isolated using the QIAamp DNA Mini Kit (Qiagen, Hilden, Germany), following the manufacturer's instructions according to DNA purification protocol for blood/body fluids. Quantification of isolated DNA was performed using a Qubit Fluorometer (Invitrogen, Life Technologies, Grand Island, NY, USA). The V3 sequence of the 16*S* ribosomal RNA (16*S* rRNA) gene was amplified using V3 region-specific primers (forward primer; CCTACGGGNGGCWGCAG: reverse primer; ATTCCGCGGCTGGCA) (Lane, 1991). The V3 region sequences were sequenced on the Illumina Miseq system.

### Sequence Analysis

Filtering steps were performed to remove low-quality reads. The filtered 16*S* rRNA gene sequencing data were analyzed by Quantitative Insights Into Microbial Ecology (QIIME) (V1.9) (Caporaso et al., 2010). The reads were clustered into operational taxonomic units (OTUs) using UCLUST with 97% similarity. The sequences were taxonomically assigned based on the Greengenes Database (DeSantis et al., 2006). Alpha-diversity analyses were performed using alpha diversity. The Shannon index, Simpson diversity index, the Abundance-based Coverage Estimator (ACE), Chao1 index, and Good's coverage index were analyzed. Differences in alpha diversity (single index) were compared using the *t*-test controlled with 10^3^ Monte Carlo permutations. Principal coordinate analysis (PCoA) was calculated based on unweighted and weighted UniFrac distance matrices to observe the differences between individuals or groups; PC1 represents the principal coordinate component that can explain the variation in data as much as possible, and PC2 is the principal coordinate component that explains the largest proportion in the remaining degree of variation. The most important differences in relative abundances among the groups were analyzed using linear discriminant analysis (LDA) effect size (LEfSe) with the throat cancer-related bacteria. Bacterial taxa with LDA scores >4 and a *p*-value <0.05 were considered strikingly enriched. Taxa with LDA scores >2 and a *p*-value <0.05 were regarded to be significantly different.

### Real-Time Quantitative Polymerase Chain Reaction

The abundance of *Aggregatibacter, Pseudomonas, Bacteroides, Faecalibacterium*, and *Ruminiclostridium* was investigated in the healthy sample, throat cancer, and polyp groups by real-time quantitative polymerase chain reaction (qPCR). A total of 10 saliva samples were randomly selected from each group, and real-time qPCR was performed independently on an ABI 7500 Genetic Analyzer (Thermo Fisher Scientific Inc., Carlsbad, CA, USA).

The PCR profile was as follows: 50°C for 2 min, 95°C for 2 min, 40 cycles of 95°C for 15 s, and 60°C for 1 min. A total of 50 ng of extracted DNA and 0.5 μl of each primer (10 pmol) were added to 10 μl of PowerUP SYBR Green Master Mix (Thermo), and the volume was adjusted to 20 μl following the addition of distilled water. All assays were performed in triplicate for each sample. A negative control consisting of DNA-free water was used for each assay. The real-time qPCR data analysis was performed with the ABI 7500 Software with manually set threshold. The Ct value was <35 for targeted samples and >38 for negative control. Finally, ΔCt was calculated according to the method ΔCt = Ct_target_-Ct_control_ and relative abundances = POWER(2, –ΔCt). Primers used for the quantification of *Pseudomonas, Faecalibacterium*, and *Bacteroides* and the universal primer in the study were referred from previous publications. The primers of *Pseudomonas* (forward primer: 5′-CTACGGGAGGCAGCAGTGG-3′, reverse primer: 5′-TCGGTAACGTCAAAACAGCAAAGT-3′) (Purohit et al., 2003), *Faecalibacterium* (forward primer: 5′-CTAACTACGTGCCAGCAGCC-3′, reverse primer: 5′-GCCTTCGCCACTGGTGTTCC-3′) (Duan et al., 2016), *Bacteroides* (forward primer: 5′-GAGAGGAAGGTCCCCCAC-3′, reverse primer: 5′-CGCTACTTGGCTGGTTCAG-3′) (Guo et al., 2010), and the universal primer (forward primer: 5′-ACTCCTACGGGAGGCAGCAG-3′, reverse primer: 5′-ATTACCGCGGCTGCTGG-3′) (Fierer et al., 2005) were used, respectively. The sequence encoding 16*S* rRNA was retrieved from the U.S. National Center for Biotechnology Information (NCBI) GeneBank database to design primers for specific detection of *Ruminiclostridium* (accession nos. NR_026104, NR_026102, NR_112037, and NR_117165, NR_117164) (forward primer: 5′-GGTGAGTAACGCGTGGGTAA-3′, reverse primer: 5′-AACTAGCTAATCGGACGCGG-3′) and *Aggregatibacter* (accession nos. CP012067 and CP012959) (forward primer: 5′-ACGGGTGAGTAATGCTTGG-3′, reverse primer: 5′-GAGATCGTCGGCTTGGTAGG-3′). The conserved sequences were selected using ClustalW program, and the primers were designed from this highly conserved region.

## Results

### Oral Microbiota Profiles Generated by 16*S* rRNA Sequencing

**Figure 1:**
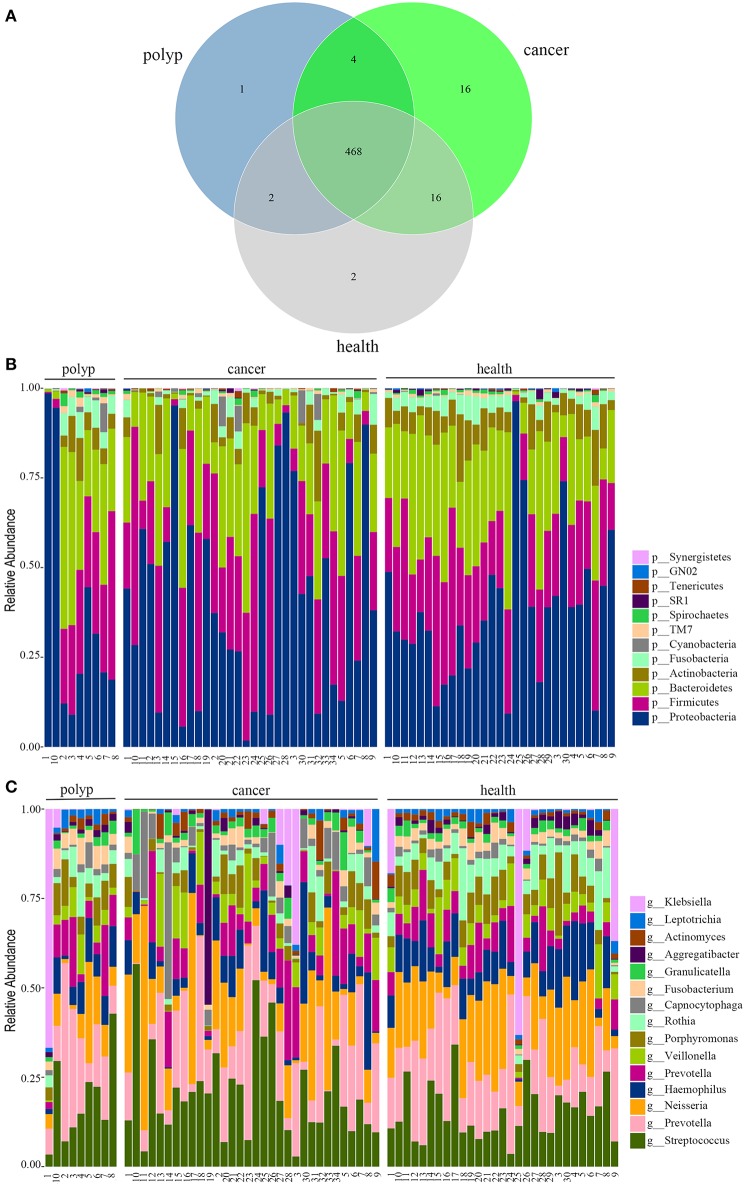
The oral microbiota profiles of samples from throat cancer patients, vocal cord polyp patients, and healthy individuals. **(A)** A Venn diagram comparing the operational taxonomic unit (OTU) distribution among the throat cancer, vocal cord polyp, and normal control groups. **(B)** The relative abundance of the dominant phyla in the throat cancer, vocal cord polyp, and normal control groups. **(C)** The relative abundance of the dominant genera in the throat cancer, vocal cord polyp, and normal control groups.

In this study, 32 patients with throat cancer (17 patients with hypopharyngeal cancer and 15 patients with laryngeal cancer), nine patients with a vocal cord polyp, and 29 healthy individuals (controls) were enrolled (sample information is in Supplementary Table 1). Subjects in different groups were similar with respect to age, race, and body mass index (BMI). The subjects had no history of tobacco or alcohol use. The saliva microbiota sequences were analyzed by barcoded 16*S* rRNA next-generation sequencing. After filtering poor-quality reads, we collected an average of 138,153 clean reads and 223 OTUs per sample (Supplementary Table 1). We identified 504 OTUs in the throat cancer group, 475 OTUs in the vocal cord polyp group, and 488 OTUs in the normal control group (shown in Figure 1A). Additionally, 468 OTUs were shared among these groups, and 16, 1, and 2 unique OTUs were observed for the throat cancer, vocal cord polyp, and normal control groups, respectively (Supplementary Table 2).

The total oral microbiota OTU composition was found similar with that of a previous study in the Human Oral Microbiome Database (HOMD) (Chen et al., 2010), and the dominant phyla and genera are shown in Figures 1B,C. The class, order, and family profiles for all samples are shown in Supplementary Figure 1. The most prevalent phyla among the throat cancer, vocal cord polyp, and normal healthy groups were *Proteobacteria, Bacteroidetes, Firmicutes, Actinobacteria*, and *Fusobacteria* in the subjects analyzed, while *Streptococcus, Klebsiella, Neisseria, Prevotella, Haemophilus, Capnocytophaga, Veillonella, Porphyromonas*, and *Rothia* were among the most prevalent genera.

### Variations in Oral Microbiome Composition Among Different Groups

**Figure 2:**
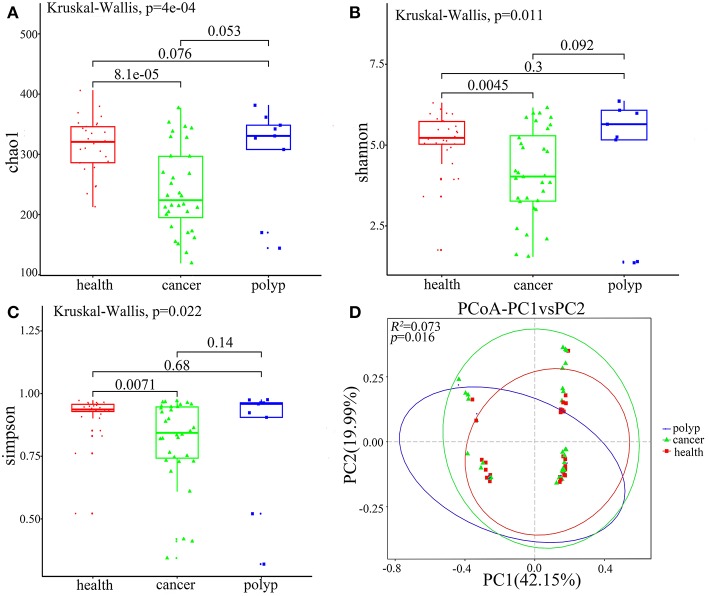
Alpha and beta diversity for the microbial community of samples from the throat cancer patients, vocal cord polyp patients, and normal control group. **(A–C)** Alpha-diversity analysis with different parameters. Chao1 **(A)**, Shannon **(B)**, and Simpson's reciprocal index **(C)**. **(D)** Principal coordinate analysis (PCoA) plots of the unweighted UniFrac distance matrix. Each symbol represents a sample. Green squares represent healthy control patients, blue triangles represent patients with throat cancer, and red circles represent patients with vocal cord polyp. The associated differences are explained in parentheses on the axes. PC1 explained 42.15% of total variation in observed variation, and PC2 explained 19.99% of the remaining variation.

Alpha diversity and beta diversity were evaluated to compare diversity in the oral microbiome among the different groups. The samples from the throat cancer patients exhibited remarkably reduced microbial diversity indices compared with the healthy control cases (*P* = 8.1e-05, 0.0045, and 0.0071 for the Chao1, Simpson, and Shannon indices, respectively) (Figures 2A–C). Beta diversity was calculated by PCoA. Following analysis of unweighted distance matrices (*P* = 0.016), we observed that the microbiota profile of patients with throat carcinoma was significantly different from that of the other groups (Figure 2D).

**Figure 3:**
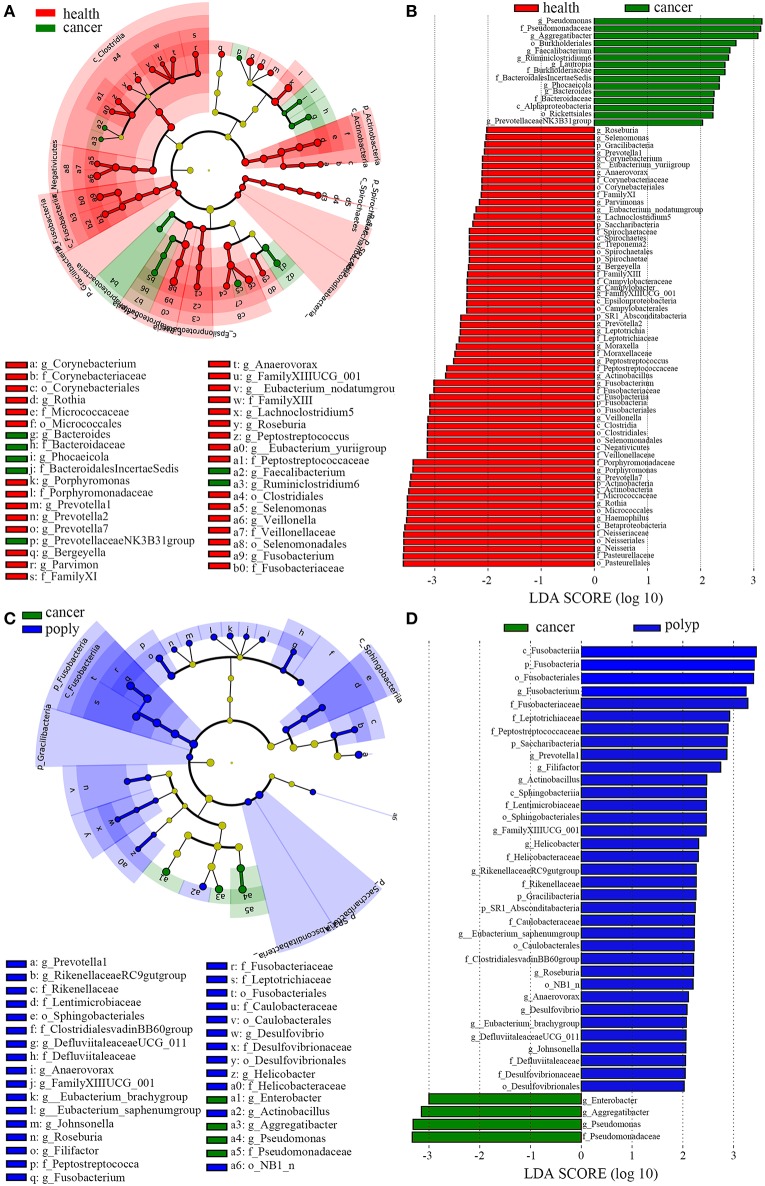
A linear discriminant analysis (LDA) was performed using linear discriminant analysis effect size (LEfSe) with three levels of clinical discrimination categories. **(A)** Cladogram representation of the oral microbiota taxa in throat cancer patients vs. healthy control individuals. **(B)** The specific microbiota taxa in throat cancer patients vs. healthy control individuals. Green indicates taxa enriched in the healthy control individuals, and red indicates taxa enriched in throat cancer patients. **(C)** Cladogram representation of the oral microbiota taxa in the throat cancer vs. the vocal cord polyp group. **(D)** The specific microbiota taxa in throat cancer patients vs. vocal cord polyp patients. Green indicates taxa enriched in throat cancer patients, and red indicates taxa enriched in vocal cord polyp patients.

LEfSe analysis (Segata et al., 2011) was also performed to compare the salivary microbial community structure of patients with throat cancer to that of vocal cord polyp patients or normal control individuals. In total, 76 taxa differed between the throat cancer group and the healthy control group (LDA scores were >2); 15 taxa were significantly enriched in the throat cancer group (Figures 3A,B). One class (*Alphaproteobacteria*), two orders (*Rickettsiales* and *Burkholderiales*), four families (*Bacteroidaceae, Bacteroidales Incertae_Sedis, Burkholderiaceae*, and *Pseudomonadaceae*), and eight genera (including *Prevotellaceae*NK3B31group, *Bacteroides, Phocaeicola, Lautropia, Ruminiclostridium6, Faecalibacterium, Aggregatibacter*, and *Pseudomonas*) were significantly enriched in the throat cancer group compared with the normal control group. Compared to the vocal cord polyp group, the *Pseudomonadaceae* family and genera *Pseudomonas, Aggregatibacter*, and *Enterobacter* were significantly enriched in the throat cancer group (Figures 3C,D).

### Validation and Identification of Diagnostic Markers for Throat Cancer

**Figure 4:**
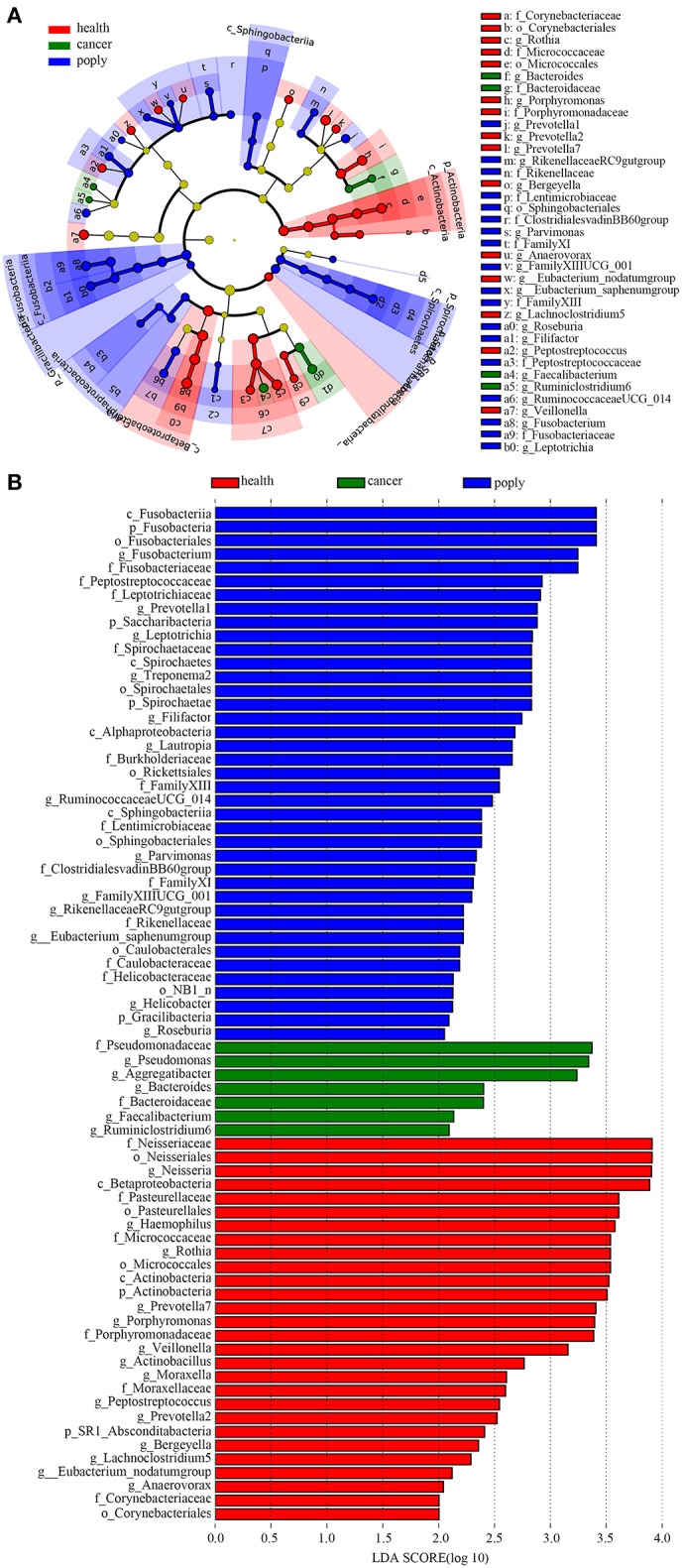
Identification of diagnostic markers for throat cancer following LDA of throat cancer and microbial taxa. **(A)** Cladogram representation of the oral microbiota taxa in the throat cancer, vocal cord polyp, and normal control groups. **(B)** Specific microbiota taxa in throat cancer patients following LDA. Green indicates taxa enriched in the throat cancer group, red indicates taxa enriched in the vocal cord polyp control group, and blue indicates taxa enriched in the healthy group.

In order to identify potentially useful markers for the diagnosis of throat cancer, an LEfSe analysis was performed to compare patients with throat cancer to vocal cord polyp patients and normal healthy individuals. A total of 74 taxa were identified with LDA scores >2. Two families (*Pseudomonadaceae* and *Bacteroidaceae*) and five genera (*Aggregatibacter, Pseudomonas, Bacteroides, Faecalibacterium*, and *Ruminiclostridium*) were significantly enriched in the throat cancer group (Figures 4A,B).

**Figure 5:**
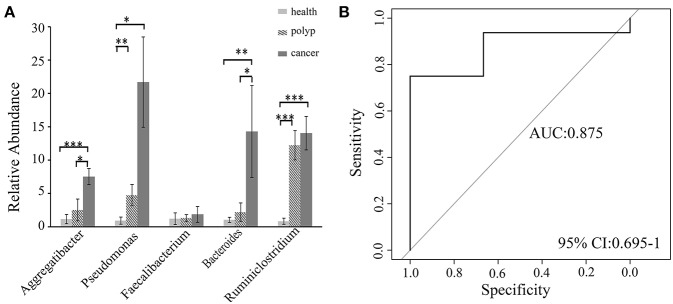
Validation of the potential throat markers for throat cancer diagnosis. **(A)** Quantitative polymerase chain reaction (qPCR) quantification analysis of *Pseudomonas, Aggregatibacter, Bacteroides, Faecalibacterium*, and *Ruminiclostridium* in the throat cancer group, vocal cord polyp group, and healthy control group; ^*^P < 0.05, ^**^P < 0.01, ^***^P < 0.001. **(B)** Receiver operating characteristic (ROC) curve analysis for throat cancer with the predictive bacterial biomarkers (*Pseudomonas, Aggregatibacter, Bacteroides, and Ruminiclostridium*). ROC curve analysis for throat cancer with the predictive bacterial biomarkers.

A non-parametric Kruskal–Wallis test was performed to confirm differences in abundances among the throat cancer, vocal cord polyp, and normal healthy groups for each bacterial taxon. The analysis confirmed that *Pseudomonas, Aggregatibacter, Bacteroides, Faecalibacterium*, and *Ruminiclostridium6* were prominently ample in the throat cancer group (*P* < 0.05) (Table 1). Real-time PCR was performed to further analyze the relative abundance of *Pseudomonas, Aggregatibacter, Bacteroides, Faecalibacterium*, and *Ruminiclostridium6* among the different patient groups. The relative abundance of *Pseudomonas, Aggregatibacter, Bacteroides*, and *Ruminiclostridium6* was significantly high among the throat cancer group (*P* < 0.05) (Figure 5A). Receiver operating characteristic (ROC) curve analysis was subsequently performed with the putative throat cancer markers (*Pseudomonas, Aggregatibacter, Bacteroides*, and *Ruminiclostridium6*). As revealed by the area under the curve [AUC = 0.875, 95% confidence interval (CI): 0.695–1], this model exhibited a robust and statistically significant diagnostic accuracy (Figure 5B).

**Table 1:** The difference in abundance for each bacterial taxon in samples from the throat cancer patients, vocal cord polyp patients, and normal control group.

**Genera**	***p*-value**	**Relative abundance mean**		
		**Health**	**Cancer**	**Polyp**
*Corynebacterium*	0.007	0.002	0.002	0.002
*Corynebacterium*1	0.041	0	0	0
*Rothia*	0	0.055	0.021	0.034
*Bacteroides*	0.028	0	0.002	0.001
*Phocaeicola*	0.001	0	0	0
*Porphyromonas*	0.003	0.051	0.024	0.039
*Prevotella*1	0.003	0.002	0.001	0.009
*Prevotella*2	0.001	0.006	0.003	0.005
*Prevotella*7	0.041	0.078	0.055	0.057
*Prevotellaceae*NK3B31group	0.022	0	0	0
*Rikenellaceae*RC9gutgroup	0.022	0.001	0	0.002
*Bergeyella*	0.004	0.004	0.002	0.002
*Defluviitaleaceae*UCG011	0.027	0	0	0.001
*Parvimonas*	0.012	0.003	0.001	0.003
*Anaerovorax*	0	0.001	0	0
*Family*XIIIUCG001	0.008	0	0	0
*Eubacteriumnodatumgroup*	0.048	0.003	0.002	0.002
*Eubacteriumsaphenumgroup*	0.012	0	0	0.001
*Johnsonella*	0.018	0.001	0.001	0.001
*Lachnoclostridium5*	0.01	0.006	0.004	0.004
*Roseburia*	0.005	0	0	0
*Peptococcus*	0.022	0	0	0.001
*Filifactor*	0	0.002	0.001	0.006
*Peptostreptococcus*	0.005	0.006	0.002	0.004
*Eubacteriumyuriigroup*	0	0.001	0.001	0.001
*Faecalibacterium*	0.005	0	0	0
*Ruminiclostridium*6	0.022	0	0	0
*Ruminococcaceae*UCG014	0.024	0.003	0.002	0.005
*Bulleidia*	0.045	0	0	0
*Veillonella*	0.026	0.044	0.033	0.029
*Fusobacterium*	0.001	0.023	0.012	0.029
*Leptotrichia*	0.023	0.012	0.008	0.014
*Lautropia*	0.007	0.005	0.005	0.008
*Neisseria*	0.016	0.142	0.114	0.063
*Helicobacter*	0.036	0	0	0
*Actinobacillus*	0	0.009	0.003	0.004
*Aggregatibacter*	0.001	0.012	0.016	0.009
*Haemophilus*	0.002	0.081	0.043	0.048
*Moraxella*	0.026	0.004	0.001	0
*Pseudomonas*	0.001	0	0.016	0
*CandidatusSaccharimonas*	0.007	0.002	0.001	0.002
*Treponema*2	0.017	0.005	0.004	0.01
*Acholeplasma*	0.016	0.001	0.001	0.001

## Discussion

Relevance between oral microbiome and throat cancer has been reported in a previous study (Hayes et al., 2018). The relative abundance of *Corynebactrium* and *Kingella* was associated to the risk of laryngeal tumor with smoking usage. As commonly known, smoking was the major risk factor for the incidence of throat cancer (Lubin et al., 2010; Anantharaman et al., 2011; Amma et al., 2013). It has been reported that smoking could influence the oral microbiota colonization and lead to upper respiratory disease (Charlson et al., 2010; Yu et al., 2017). Smoking may affect the correlation of the oral bacteria with laryngeal cancer, and the correlation needs to be studied independently.

In the current study, we identified the oral bacterial markers for diagnosis of throat cancer, using a cohort of subjects with either hypopharyngeal carcinoma or laryngeal cancer, vocal cord polyp, and normal healthy individuals. All the subjects had no history of smoking. This result revealed, for the first time, that the oral bacterial features of throat cancer patients were different from those of vocal cord polyp patients and normal healthy individuals. The PCoA analysis of unweighted distance matrices revealed that the microbiota profile for the throat cancer group was significantly different from that of the healthy control group. In addition, we also observed significant reductions in microbiota diversity and richness in the throat tumor samples. Statistical analysis revealed that the Chao1 (*P* = 8.1e-05), Simpson (*P* = 0.0045), and Shannon (*P* = 0.0071) indices were significantly reduced in the saliva samples of tumor patients. Previous studies have shown that reductions in the microbial diversity of the human microbiome are linked to cancer occurrence (Schmidt et al., 2014; Gao J. et al., 2018). The associated reduction in bacterial diversity reflected the altering of the abundance in certain bacteria and their metabolic pathways. The “oncogenic bacteria” have been reported to promote tumorigenesis through the indirect inflammatory effects and/or the direct epigenetic mechanism (Sauid and Lois, 2015; Atarashi et al., 2017; Chung et al., 2018). The oral “oncogenic bacteria” associated with throat cancer may migrate with the saliva flow and colonize in the throat by swallowing, which will induce cancer indirectly or directly as a consequence.

Through the LDA analysis on throat cancer vs. normal group, throat cancer vs. vocal polyp, and throat cancer vs. the other two matching groups, bacterial taxa, which might cause throat cancer, were identified. We found that the genera *PrevotellaceaeNK3B31group, Bacteroides, Phocaeicola, Lautropia, Ruminiclostridium, Faecalibacterium, Aggregatibacter*, and *Pseudomonas* were significantly enriched in the throat cancer group. These taxa associated only with throat cancer, and they may play important roles in the occurrence of cancer. Compared to the vocal cord polyp group, *Pseudomonas, Aggregatibacter*, and *Enterobacter* were significantly enriched in the throat cancer group. In the throat cancer groups, *Pseudomonadaceae, Bacteroidaceae, Aggregatibacter, Pseudomonas, Bacteroides, Faecalibacterium*, and *Ruminiclostridium* had the highest LDA scores. Increase in the abundance of *Pseudomonas, Aggregatibacter, Bacteroides*, and *Ruminiclostridium* in the throat cancer group (*P* < 0.05) was also observed by using the Kruskal–Wallis test and real-time qPCR. However, the LDA analysis had shown some anomalies for several microbial groups. Some bacterial taxa associated with several groups. For example, *Rickettsiales* and *Lautropia* were related with throat cancer, and *Spirochaetales* was health-associated according to the LDA analysis on throat cancer vs. health group, while they appeared as associated with vocal polyps on the basis of LDA analysis on the three groups. These results indicated that these bacteria may not play important roles in the associated groups.

The ROC models were constructed using the aforementioned obviously different taxa with random combination (data not shown). The ROC model with the taxa *Aggregatibacter, Pseudomonas, Bacteroides*, and *Ruminiclostridium* performed better than with the other taxa in the ROC model analysis. It proved to be robust with statistically significant diagnostic accuracy and had an acquired accuracy of 87.5%. These results indicated that *Pseudomonas, Aggregatibacter, Bacteroides*, and *Ruminiclostridium* may have a significant correlation with throat cancer. *Aggregatibacter, Pseudomonas*, and *Bacteroides* were reported to associate with aggressive periodontitis and oral infection (Leys et al., 2002; Souto et al., 2014; Cordero and Varela-Calviño, 2018). Periodontitis and oral infection are risk factors for head and neck cancer (Xian-Tao et al., 2013a,b, 2015; Moritani et al., 2015). *Pseudomonas*, a gram-negative bacterium with carbon degradation activity, was the most common pathogen for hospital infection (Hota et al., 2009; Decker and Palmore, 2014; Hung et al., 2016). It can invade the epithelium and induce immune responses through activation of protein kinase C alpha (PKCα), c-Jun *N*-terminal kinase (JNK), extracellular-regulated protein kinases (ERK1/2), nuclear factor kappa-light-chain-enhancer of activated B cells (NF-κB), and/or glutamic acid–leucine–arginine–positive CXC chemokines (Gregson et al., 2013; Chiang-Wen et al., 2018; Curran et al., 2018). *Aggregatibacter* was associated with aggressive periodontitis (Ando et al., 2010), which can induce inflammatory response through cytolethal distending toxin (CDT), leukotoxin, and lipopolysaccharide (LPS) (Herbert et al., 2016). *Bacteroides fragilis* has been verified to correlate with diarrheal disease and inflammatory bowel and can induce colon carcinogenesis through indirect inflammatory effects and direct epigenetic pathways (Toprak et al., 2006; Chung et al., 2018). It can cleave catenins and lead activation of oncogene c-myc (Hardy et al., 2000). As a conclusion, *Aggregatibacter, Pseudomonas*, and *Bacteroides* may have individual or combined inducing effects on tumorigenesis through indirect inflammatory and (or) direct epigenetic pathway. Further study is required to confirm the relevant mechanism between tumorigenesis and infection of these bacteria.

In summary, we performed a comprehensive comparison of the oral bacterial taxa among throat cancer patients, vocal cord polyp patients, and healthy individuals. The study revealed different oral microbiota profiles among the analyzed groups. The analysis also detected reduced diversity and richness in the oral bacterial community in throat cancer patients, indicating that oral bacteria may play an important role in the occurrence of throat cancer. A total of four genera, *Pseudomonas, Aggregatibacter, Bacteroides*, and *Ruminiclostridium*, were identified significantly associated with throat cancer. However, the pathogenesis that cause this phenomenon still needs to be clarified, and large cohort studies are required to verify the results from this study.

## Ethics Statement

The study was approved by ethics committee of Beijing Tongren Hospital, Capital Medical University (approval number TRECKY2018-007). All participants upon recruitment signed informed consent.

## Author Contributions

LW, ZH, WG, and TS conceived and designed the study and contributed to the writing of the manuscript. GY and WG helped in the data acquisition. LW, GY, YG, YZ, MZ, YL, and PS analyzed and interpreted the data. All the authors read and approved the final manuscript.

### Conflict of Interest Statement

LW, YG, YZ, MZ, YL, PS, and TS were employed by Beijing Cheer Land Biotechnology Co., Ltd., CL Investment Group. The remaining authors declare that the research was conducted in the absence of any commercial or financial relationships that could be construed as a potential conflict of interest.

## Supplementary Material

Table S1 Sample information in the study.

Table S2 Unique OTUs in throat cancer group, vocal cord polyp group, and normal control group.

Supplementary Figure 1 The oral microbiota profiles of samples from throat cancer patients, vocal cord polyp patients, and healthy individuals. **(A)** The relative abundance of the dominant classes in the throat cancer, vocal cord polyp, and normal control groups. **(B)** The relative abundance of the dominant orders in the throat cancer, vocal cord polyp, and normal control groups. **(C)** The relative abundance of the dominant families in the throat cancer, vocal cord polyp, and normal control groups.
