# Supplementary material for: Variations in Oral Microbiota Composition Are Associated With a Risk of Throat Cancer
Source: Front Cell Infect Microbiol. 2019 Jul 3;9:205. doi: 10.3389/fcimb.2019.00205 (PMC6618584; doi:10.3389/fcimb.2019.00205)
Supplement: Table S1 — Sample information in the study. [file Table_1.DOCX]

Supplementary table 1. Sample information in the study.

| Study_ID | Ethnicity | Gender | Age (years) | Height (m) | Weight (kg) | BMI (kg/m^2^) | Clinical stage | Disease | reads | | OTUs |
| --- | --- | --- | --- | --- | --- | --- | --- | --- | --- | --- | --- |
|  |  |  |  |  |  |  |  |  | Raw reads | clean reads |  |
| polyp1  polyp2  polyp3  polyp4  polyp5  polyp6  polyp7  polyp8  polyp9  cancer1  cancer2  cancer3  cancer4  cancer5  cancer6  cancer7  cancer8  cancer9  cancer10  cancer11  cancer12  cancer13  cancer14  cancer15  cancer16  cancer17  cancer18  cancer19  cancer20  cancer21  cancer22  cancer23  cancer24  cancer25  cancer26  cancer27  cancer28  cancer29  cancer30  cancer31  cancer32  health1  health2  health3  health4  health5  health6  health7  health8  health9  health10  health11  health12  health13  health14  health15  health16  health17  health18  health19  health20  health21  health22  health23  health24  health25  health26  health27  health28  health29 | Asian  Asian  Asian  Asian  Asian  Asian  Asian  Asian  Asian  Asian  Asian  Asian  Asian  Asian  Asian  Asian  Asian  Asian  Asian  Asian  Asian  Asian  Asian  Asian  Asian  Asian  Asian  Asian  Asian  Asian  Asian  Asian  Asian  Asian  Asian  Asian  Asian  Asian  Asian  Asian  Asian  Asian  Asian  Asian  Asian  Asian  Asian  Asian  Asian  Asian  Asian  Asian  Asian  Asian  Asian  Asian  Asian  Asian  Asian  Asian  Asian  Asian  Asian  Asian  Asian  Asian  Asian  Asian  Asian  Asian | male  male  male  male  male  male  female  male  female  male  male  male  male  male  male  male  male  male  male  male  male  male  male  male  male  male  male  male  male  male  male  male  male  male  male  male  male  male  male  male  male  male  male  male  male  male  male  male  male  male  male  male  male  male  male  male  male  male  male  male  male  male  male  male  male  female  female  female  female  female | 45  61  45  69  40  56  39  54  35  62  68  51  71  49  60  68  57  59  45  60  78  59  68  49  55  48  50  50  52  61  56  54  72  61  56  48  66  67  64  51  53  44  40  38  60  64  56  44  52  67  47  51  44  55  57  57  57  55  46  47  53  52  44  44  44  47  50  53  55  58 | 1.81  1.73  1.7  1.65  1.73  1.73  1.6  1.7  1.63  1.68  1.63  1.73  1.72  1.75  1.61  1.72  1.67  1.73  1.83  1.8  1.69  1.76  1.65  1.73  1.6  1.76  1.78  1.75  1.7  1.8  1.7  1.65  1.8  1.74  1.7  1.7  1.71  1.75  1.73  1.75  1.7  1.67  1.74  1.71  1.7  1.64  1.75  1.71  1.8  1.76  1.65  1.65  1.77  1.72  1.7  1.68  1.72  1.7  1.69  1.7  1.6  1.71  1.65  1.73  1.67  1.6  1.55  1.65  1.72  1.63 | 91  65  76  71  83  60  72  64  61  57  53  79  80  85  59  72  60  83  63  84  60  75  58  65  50  72  58  69  72  90  70  84  80  75  60  51  70  73  72  86  75  66  80  65  76  74  80  60  90  80  80  60  77  73  76  66  68  70  65  69  64  70  55  68  66.5  53  52  67.5  70  70c | 27.78  21.72  26.30  26.08  27.73  20.05  28.13  22.15  22.96  20.20  19.95  26.40  27.04  27.76  22.76  24.34  21.51  27.73  18.81  25.93  21.01  24.21  21.30  21.72  19.53  23.24  18.31  22.53  24.91  27.78  24.22  30.85  24.69  24.77  20.76  17.65  23.94  23.84  24.06  28.08  25.95  23.67  26.42  22.23  26.30  27.51  26.12  20.52  27.78  25.83  29.38  22.04  24.58  24.68  26.30  23.38  22.99  24.22  22.76  23.88  25.00  23.94  20.20  22.72  23.84  20.70  21.64  24.79  23.66  26.35 | /  /  /  /  /  /  /  /  /  IV  III  IV  III  I  IV  II  III  I  IV  II  IV  III  IV  III  I  II  IV  II  II  IV  IV  III  IV  II  IV  IV  II  III  III  II  IV  /  /  /  /  /  /  /  /  /  /  /  /  /  /  /  /  /  /  /  /  /  /  /  /  /  /  /  /  / | vocal cord polyp  vocal cord polyp  vocal cord polyp  vocal cord polyp  vocal cord polyp  vocal cord polyp  vocal cord polyp  vocal cord polyp  vocal cord polyp  hypopharyngeal carcinoma  hypopharyngeal carcinoma  hypopharyngeal carcinoma  hypopharyngeal carcinoma  hypopharyngeal carcinoma  hypopharyngeal carcinoma  hypopharyngeal carcinoma  hypopharyngeal carcinoma  hypopharyngeal carcinoma  hypopharyngeal carcinoma  hypopharyngeal carcinoma  hypopharyngeal carcinoma  hypopharyngeal carcinoma  hypopharyngeal carcinoma  hypopharyngeal carcinoma  hypopharyngeal carcinoma  hypopharyngeal carcinoma  Laryngeal cancer  Laryngeal cancer  Laryngeal cancer  Laryngeal cancer  Laryngeal cancer  Laryngeal cancer  Laryngeal cancer  Laryngeal cancer  Laryngeal cancer  Laryngeal cancer  Laryngeal cancer  Laryngeal cancer  Laryngeal cancer  Laryngeal cancer  Laryngeal cancer  health  health  health  health  health  health  health  health  health  health  health  health  health  health  health  health  health  health  health  health  health  health  health  health  health  health  health  health  health | 959473  76213  106012  15884  275272  73899  91740  71584  88053  172892  120391  451674  18382  253483  22080  1127635  740203  36896  94776  32903  160669  43506  65994  39444  14951  10242  31944  40185  59752  43914  77459  40756  66603  43691  45221  132359  70718  206085  59254  38097  11542  31101  1513685  92910  160457  15678  16401  76701  183834  149253  53408  26641  90661  41450  65424  183408  74119  37468  94137  88152  42418  56810  31741  251047  84098  153028  19432  15932  13704  148338 | 959165  74110  103129  15634  273072  73066  91253  71211  88019  171492  120254  441492  18214  252873  21612  1126978  707828  36889  94684  32686  149550  43492  65895  38012  14935  9660  31856  39366  58970  43716  76406  40540  66464  43463  45091  132030  70568  204923  58826  38045  11494  30940  1496720  92146  159455  15576  15878  74597  181768  147163  53197  26152  88754  41224  63627  182489  70757  37075  91448  87932  41822  56501  31566  228914  84032  152251  19181  15461  13534  147520 | 94  283  245  326  268  297  314  283  127  286  158  153  285  196  268  111  251  157  102  162  173  144  107  291  222  161  172  316  166  318  155  283  201  155  197  85  137  114  262  113  159  243  170  243  251  294  326  244  189  272  211  292  237  318  281  288  243  284  252  225  297  276  294  204  149  246  286  286  227  219 |
