# Supplementary figures and images for: Variations in Oral Microbiota Composition Are Associated With a Risk of Throat Cancer
Source: Front Cell Infect Microbiol. 2019 Jul 3;9:205. doi: 10.3389/fcimb.2019.00205 (PMC6618584; doi:10.3389/fcimb.2019.00205)

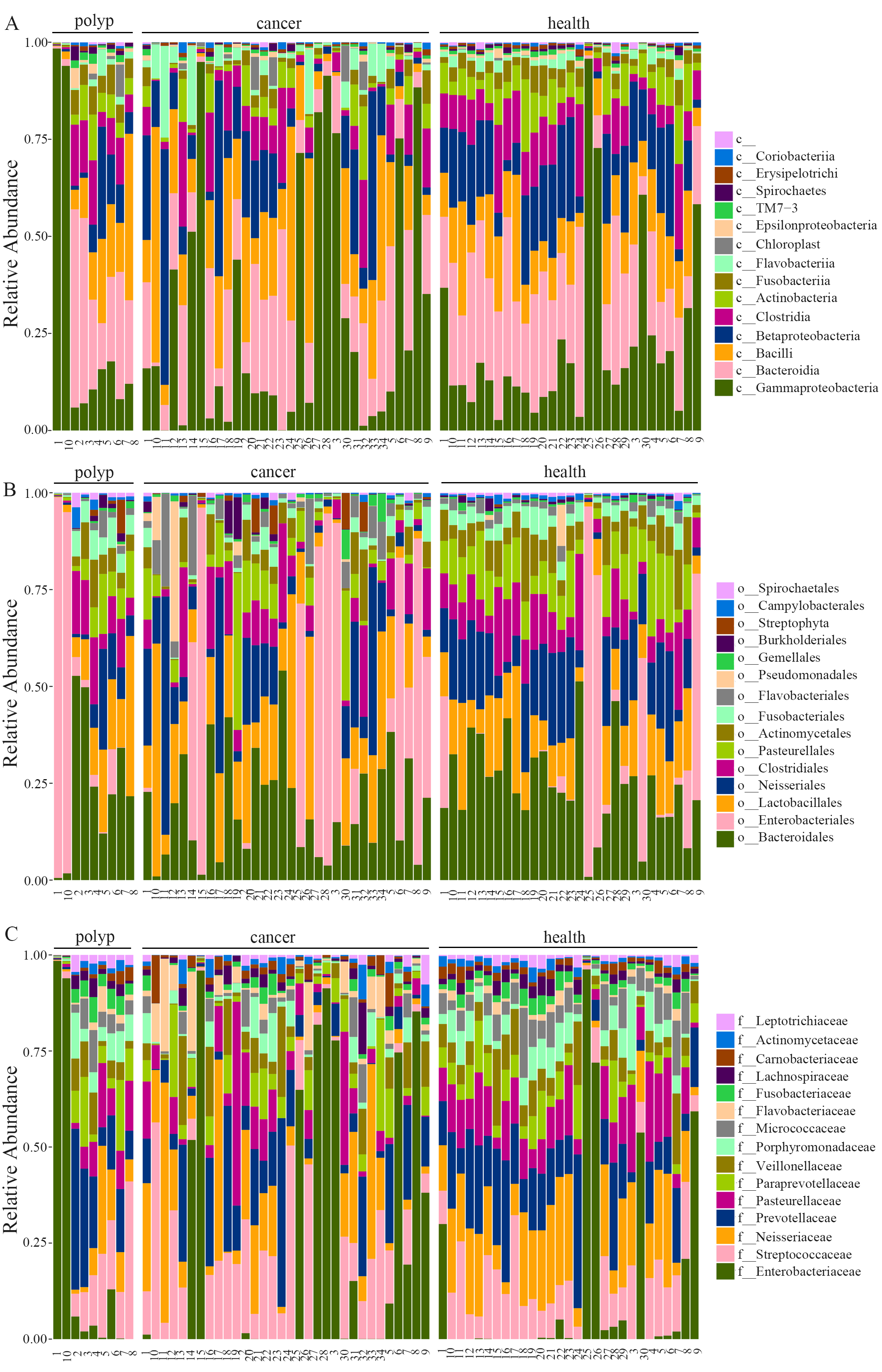

Supplement: Supplementary Figure 1 — The oral microbiota profiles of samples from throat cancer patients, vocal cord polyp patients, and healthy individuals. (A) The relative abundance of the dominant classes in the throat cancer, vocal cord polyp, and normal control groups. (B) The relative abundance of the dominant orders in the throat cancer, vocal cord polyp, and normal control groups. (C) The relative abundance of the dominant families in the throat cancer, vocal cord polyp, and normal control groups. [file Image_1.TIF]
